# Supplementary material for: Understanding West Nile virus transmission: Mathematical modelling to quantify the most critical parameters to predict infection dynamics
Source: PLoS Negl Trop Dis. 2023 May 1;17(5):e0010252. doi: 10.1371/journal.pntd.0010252 (PMC10174579; doi:10.1371/journal.pntd.0010252)
Supplement: S1 Appendix — (PDF) [file pntd.0010252.s001.pdf]

Understanding West Nile virus transmission: mathematical modelling to quantify the most critical parameters to predict infection dynamics

Elisa Fesce[1*], Giovanni Marini[2], Roberto Rosà[2,3], Davide Lelli[4], Monica Pierangela Cerioli[4], Mario Chiari[5], Marco Farioli[5], Nicola Ferrari[1,6]

[1] Department of Veterinary Medicine and Animal Science (DiVAS), Wildlife Health management & One Health Lab, Università degli Studi di Milano, Lodi (LO), Italy
[2] Research and Innovation Centre, Fondazione Edmund Mach, San Michele all'Adige, Trento (TN), Italy
[3] Center Agriculture Food Environment, University of Trento, San Michele all'Adige, Trento (TN), Italy
[4] Istituto Zooprofilattico Sperimentale della Lombardia e dell'Emilia Romagna "Bruno Ubertini" (IZSLER), Brescia (BS), Italy
[5] Regional Veterinary Authority of Lombardy, Direzione Generale Welfare, Milano (MI), Italy
[6] Centro di Ricerca Coordinata Epidemiologia e Sorveglianza Molecolare delle Infezioni, Università degli Studi di Milano, Milano (MI), Italy

* elisa.fesce@unimi.it (EF)

Epidemiological model structure

According with the scheme reported in Fig A we simulated WNV spread into the Lombardy region through the following system of differential equations:

$$
\begin{cases}
M'_S(t) = \omega(t) - \left(b \cdot p \cdot p_{BM} \cdot \dfrac{B_{Ia}(t) + B_{Ij}(t)}{B_T(t)} + \mu_M\right) \cdot M_s(t) \\
M'_E(t) = b \cdot p \cdot p_{BM} \cdot \dfrac{B_{Ia}(t) + B_{Ij}(t)}{B_T(t)} \cdot M_s(t) - (\theta_M + \mu_M) \cdot M_E(t) \\
M'_I(t) = \theta_M \cdot M_E(t) - \mu_M \cdot M_I(t) \\
B'_{Sa}(t) = -\left(b \cdot p_{MB} \cdot \dfrac{M_I(t)}{B_T(t)} + \mu_B\right) \cdot B_{Sa}(t) \\
B'_{Ea}(t) = b \cdot p_{MB} \cdot \dfrac{M_I(t)}{B_T(t)} \cdot B_{Sa}(t) - (\mu_B + \theta_B) \cdot B_{Ea} \\
B'_{Ia}(t) = \theta_B \cdot B_{Ea} - (\mu_B + \nu_B) \cdot B_{Ia} \\
B'_{Ra}(t) = \nu_B \cdot B_{Ia} - \mu_B \cdot B_{Ra} \\
B'_{Sj}(t) = \gamma \cdot B_a - \left(b \cdot p_{MB} \cdot \dfrac{M_I(t)}{B_T(t)} + \mu_{Bj}\right) \cdot B_{Sj}(t) \\
B'_{Ej}(t) = b \cdot p_{MB} \cdot \dfrac{M_I(t)}{B_T(t)} \cdot B_{Sj}(t) - \left(\mu_{Bj} + \theta_B\right) \cdot B_{Ej} \\
B'_{Ij}(t) = \theta_B \cdot B_{Ej} - \left(\mu_{Bj} + \nu_B\right) \cdot B_{Ij} \\
B'_{Rj}(t) = \nu_B \cdot B_{Ij} - \mu_{Bj} \cdot B_{Rj}
\end{cases}
$$

In the proposed system, M_S, M_E and M_I respectively represent the susceptible, exposed and infectious mosquito population, whereas B_{Sa}, B_{Ea}, B_{Ia}, and B_{Ra} represent susceptible, exposed, infectious and recovered competent adult birds, and B_{Sj}, B_{Ej}, B_{Ij}, and B_{Rj} represent susceptible, exposed infectious and recovered competent juvenile birds. Model parameters are reported in the following table:

Table A: table of parameters used in the modelling framework, with their biological explanation

Parameter	Explanation	Value	Source
μ_M	Mosquito death rate (day^{-1})	$\frac{4.61}{151.6 - 4.75 \cdot T}$	[1,2]
p_{BM}	Probability of WNV transmission from bird to mosquito per infectious bite	$\frac{e^{(-10.917+0.365 \cdot T)}}{1 + e^{(-10.917+0.365 \cdot T)}}$	[3]
θ_M	Extrinsic incubation period (day^{-1})	$0.132 + 0.0092 \cdot T$	[4]
θ_B	Intrinsic incubation period (day^{-1})	0.5	[5]
$\gamma(t)$	Avian fertility rate at day t (day^{-1})	0.5 (t $\leq$ July 20)<br>0 (t > July 20)	[6]
μ_B	Death rate of adult birds (day^{-1})	0.0015	[6]
μ_{Bj}	Death rate of juvenile birds (day^{-1})	0.0083	[6]
b	Mosquito biting rate	$f \cdot 0.122 \cdot log(T - 9)^{1.76}$	[7]
f	Fraction of bites directed to the competent bird species	*	[7], [8]
p	Avian competence	**	-
p_{MB}	Birds' susceptibility	**	-
ν_B	Avian recovery rate	**	

* Two different fraction of bites were estimated by the MCMC, one for the early season (f_1) and one for the late season (f_2)

** estimated by the MCMC

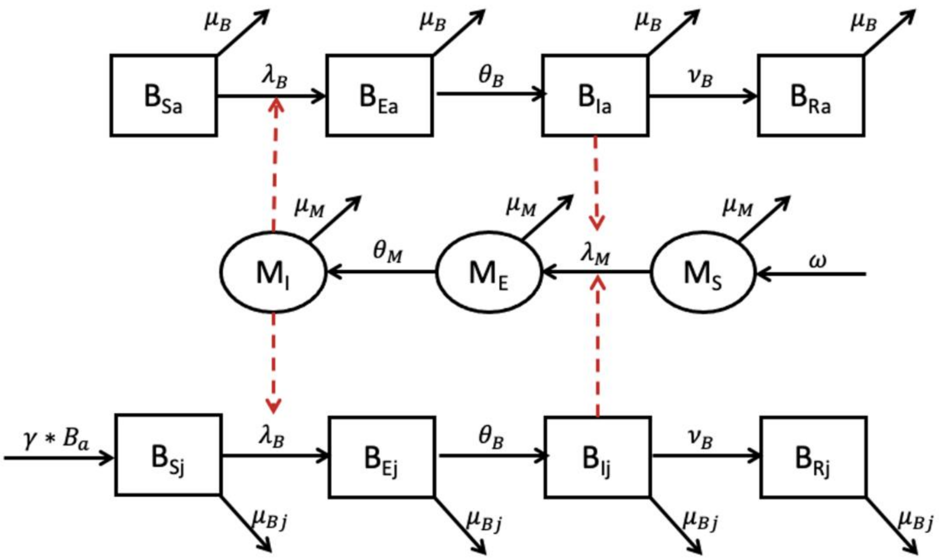

Figure A: Model scheme. Model flow chart for WNV transmission in birds (squares) and mosquitoes (circles) in an average trapped area. Compartments: B_{Sa}, B_{Ea}, B_{Ia} and B_{Ra} (B_{Sj}, B_{Ej}, B_{Ij} and B_{Rj}): adult (juvenile) susceptible, exposed, infectious and immune birds; M_S, M_E and M_I: susceptible, exposed and infectious mosquitoes. Parameters λB and λM are the forces of infection for birds and mosquitoes, respectively, and are computed as $\lambda_B = b \cdot p_{MB} \cdot \frac{M_I}{B_T}$ and $\lambda_M = b \cdot p_{BM} \cdot \frac{(B_{Ia}+B_{Ij})}{B_T}$, with B_T being the total avian population and B_a the number of adult birds.

Additional results

Model fit

The model fit was quite satisfactory, as 98% of the total (considering all years and subregions) number of weekly positive pools lay within the 95% CI predictions of the model (Fig B).

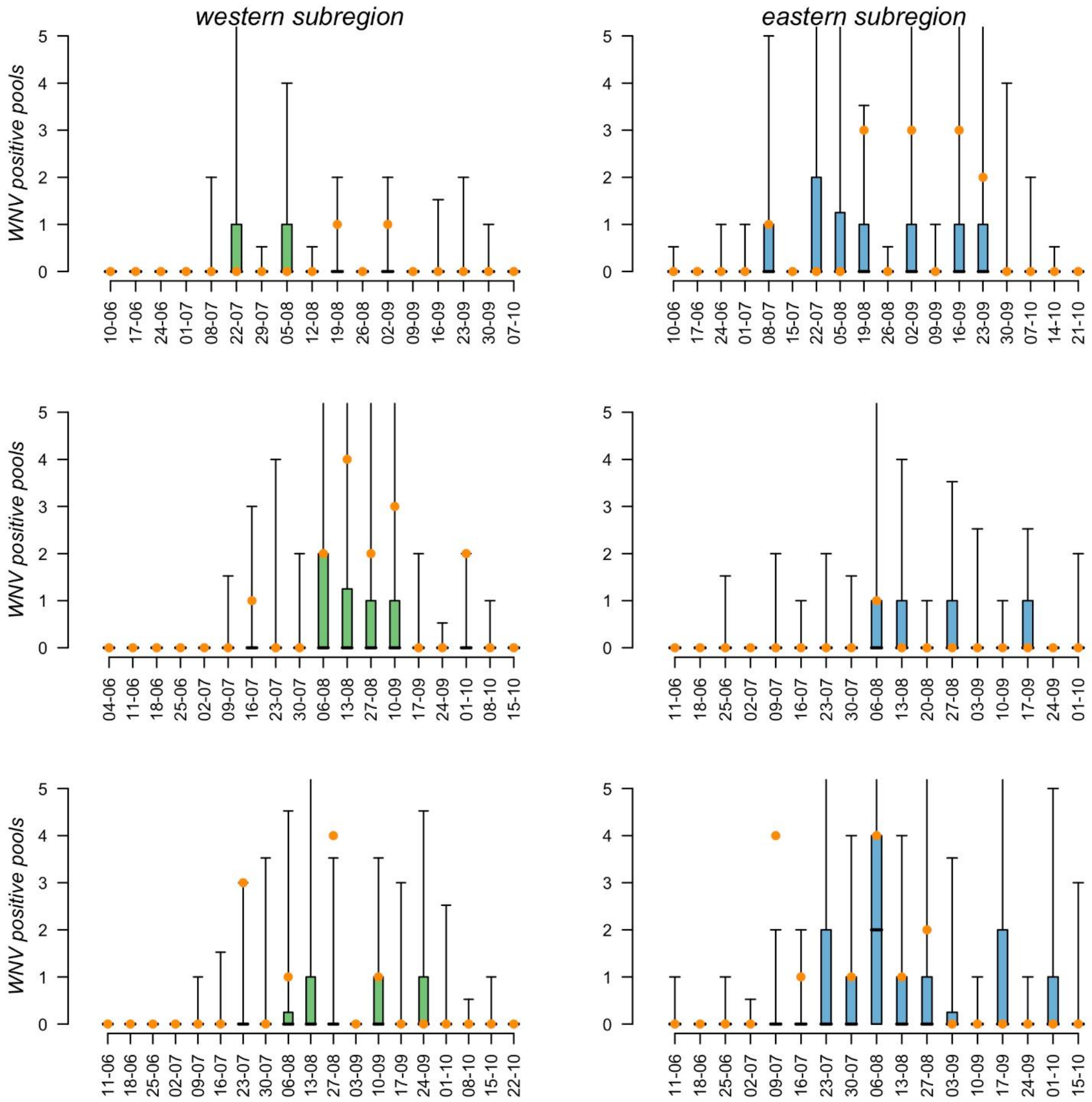

Figure B: **Model prediction fit.** Predicted number of WNV-positive pools for the three years (first line for 2016, second line for 2017 and third line for 2018) and the two areas. Orange points: observed weekly

number of WNV-positive pools; green and blue boxplots (median, 2.5 and 97.5% quantiles): the predicted distributions of positive pools per week in the western and eastern subregion respectively.

WNV spread and prevalence

We investigated WNV prevalence in mosquitoes (**Fig C**) and birds (**Fig D**) in the Lombardy region, according to model assumptions. Model simulations predict a low prevalence of WNV for the mosquito population in all years and subregions , never exceeding a daily mean prevalence of 0.231%. In all years and subregions , the lowest prevalence is shown up to July, and then we can see its increase, a peak in early/late August and a slight decrease and stabilization. The increase and decrease slopes and the timing for the prevalence peak both depend on the year and subregions considered. The lowest WNV prevalence in both mosquitoes and birds was predicted for the western subregions in 2018, with a mean mosquito prevalence of 0.053% (0-0.301% CI), and the highest was also predicted in the eastern subregions in 2018, with a mean prevalence of 0.231% (0.003-0.99% CI).

On the other hand, the avian prevalence was higher, reaching a daily mean of 8.38% (0.003-35.5% CI). It increased between June and July, reached the maximum between July and August and then slowly decreased up to October.

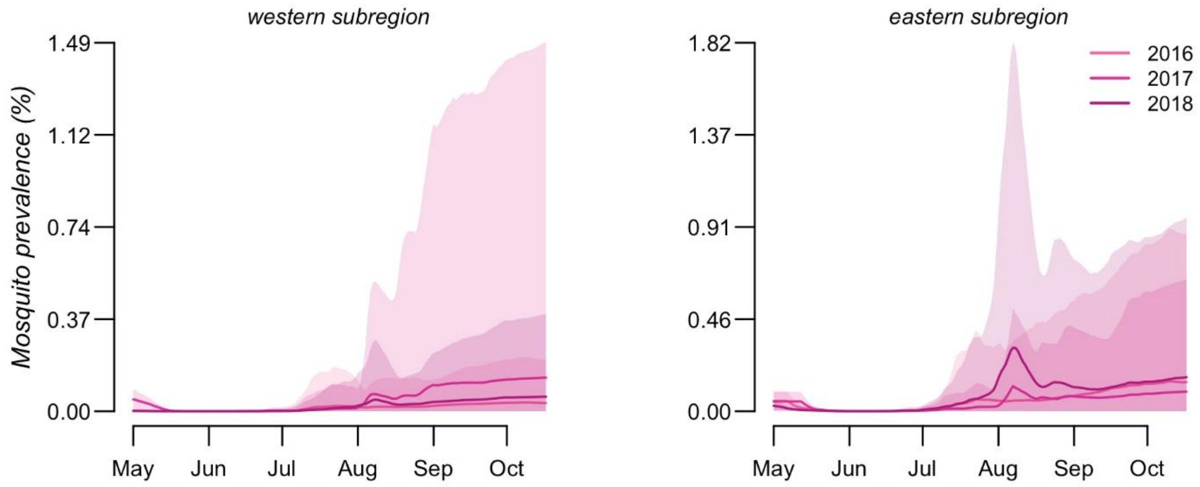

Figure C: Model predictions. Predicted WNV prevalence in mosquitoes in subregions and years.

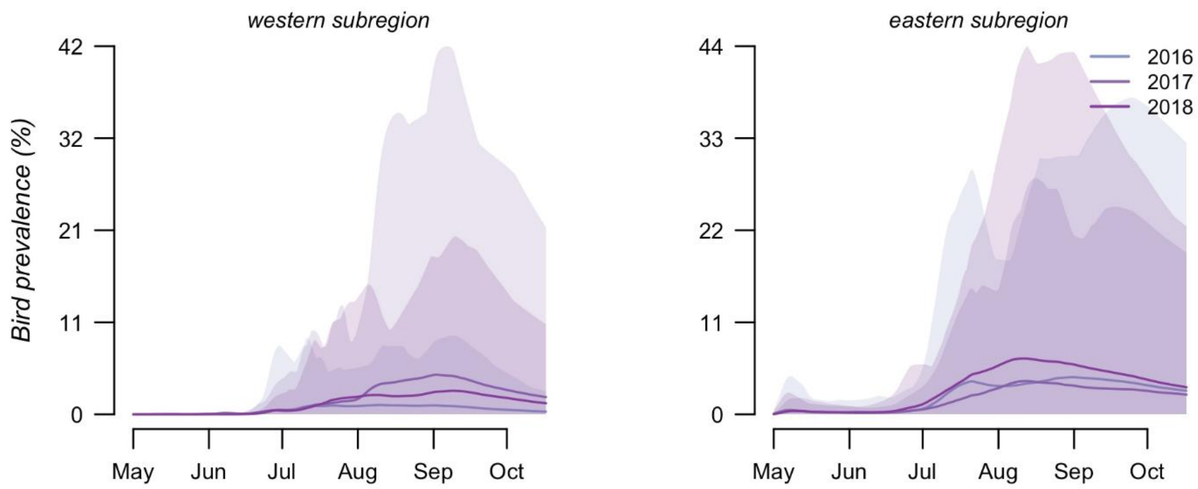

Figure D: Model predictions. Predicted WNV prevalence in the competent avian population (magpies) in subregions and years.

Parameter estimate

Posterior distributions of the free parameters are reported in Figure E. As stated in the main text, no association between the bird-related transmission parameters chosen for analysis in this context, and only the proportion of immune birds at the beginning of the first simulated season in the western and eastern subregions (i_{Bw} and i_{Be}, corr=0.98, P<0.001) and recovery rate and proportion of competent birds (ν_B and a_i, corr=-0.54, P<0.001) showed respectively positive and negative correlation (Fig F). The autocorrelation analysis instead (Fig G) pointed out some correlation for the estimated parameters.

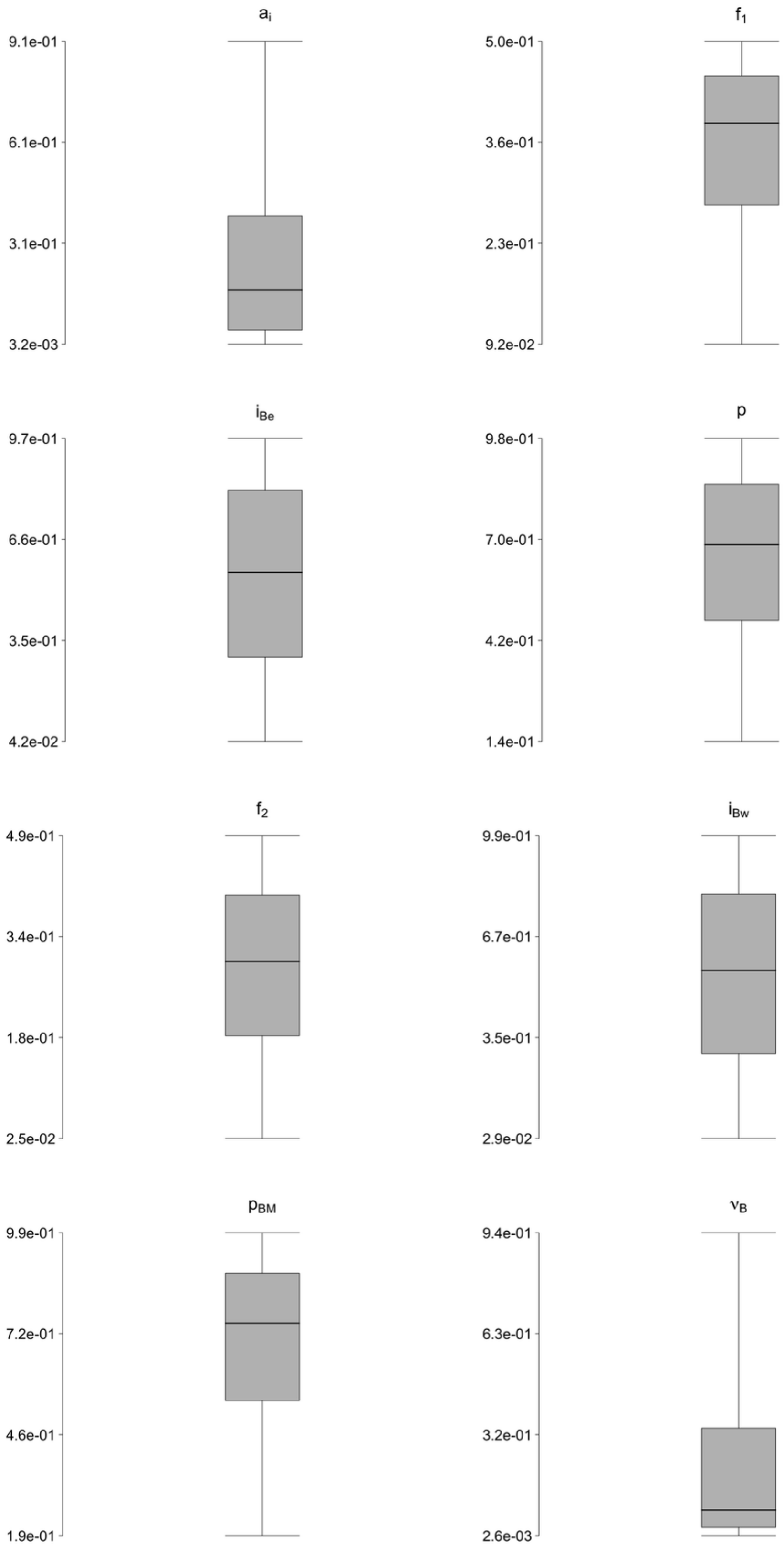

Figure E: Posterior distribution of parameters.

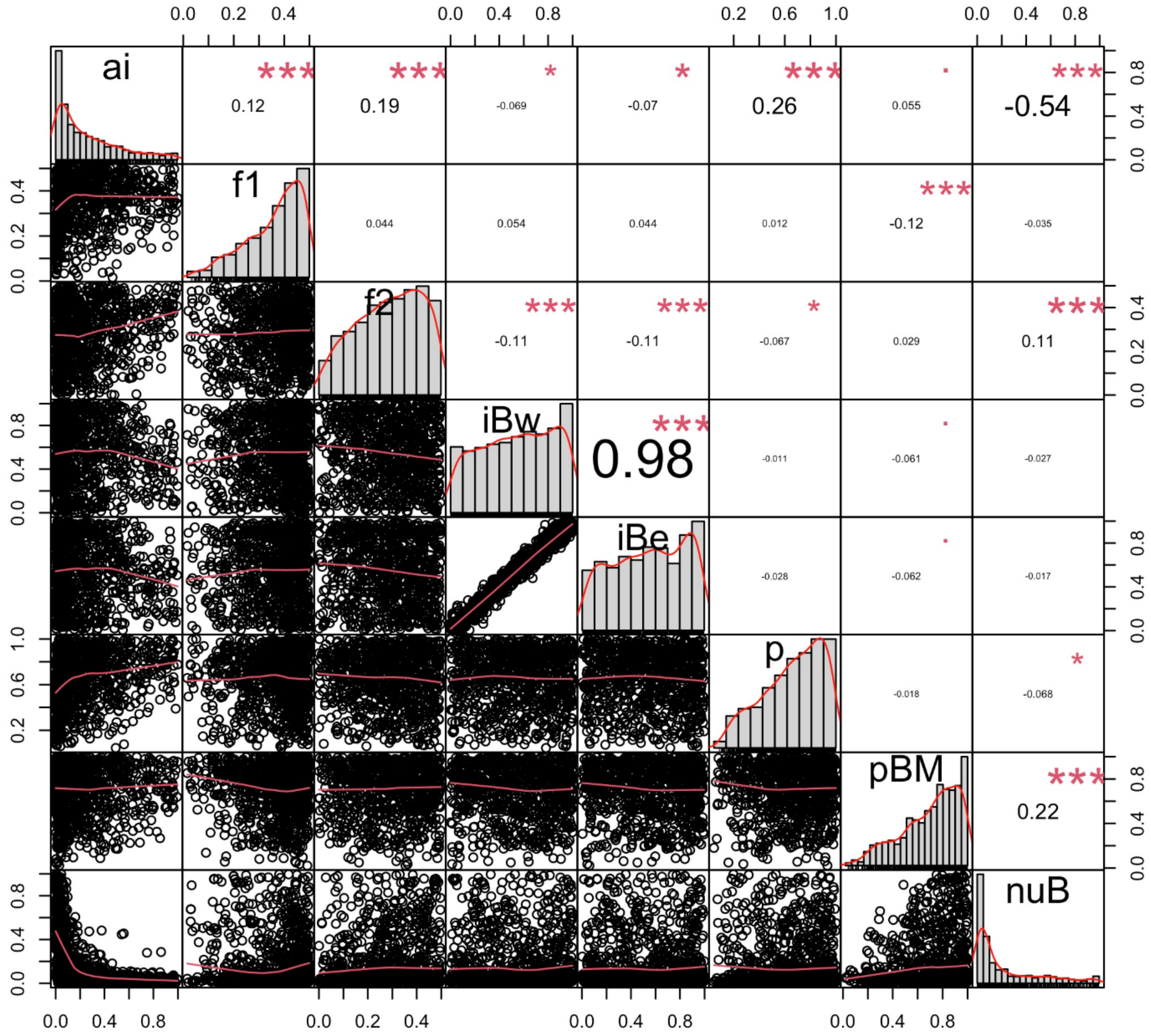

Figure F: Correlation between epidemiological parameters.

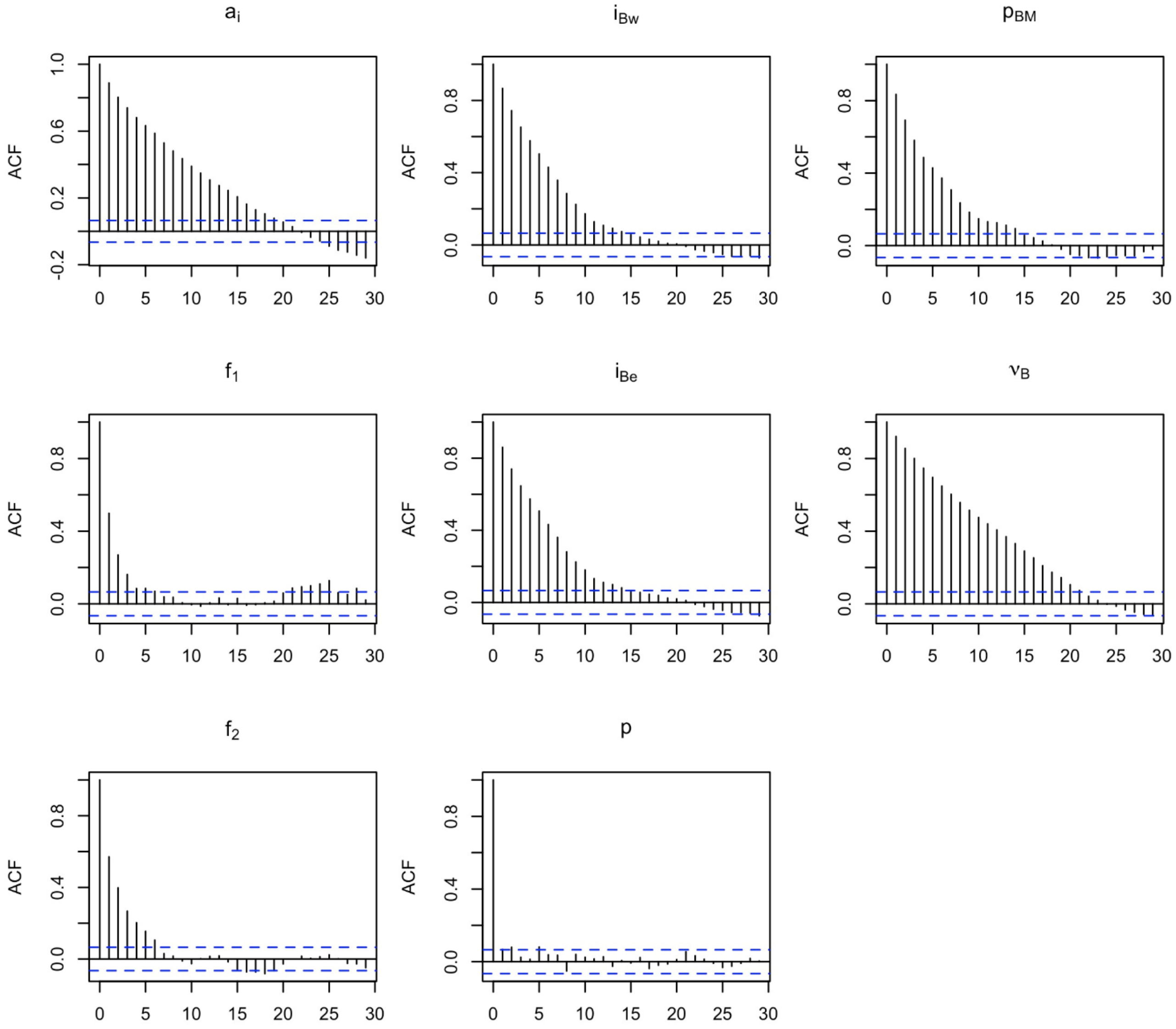

Figure G: Autocorrelation of parameters.

We carried out a pairwise perturbation analysis for the four key epidemiological parameters investigated, whose outcome is presented in Fig H. This analysis showed no combined effect of parameter perturbation.

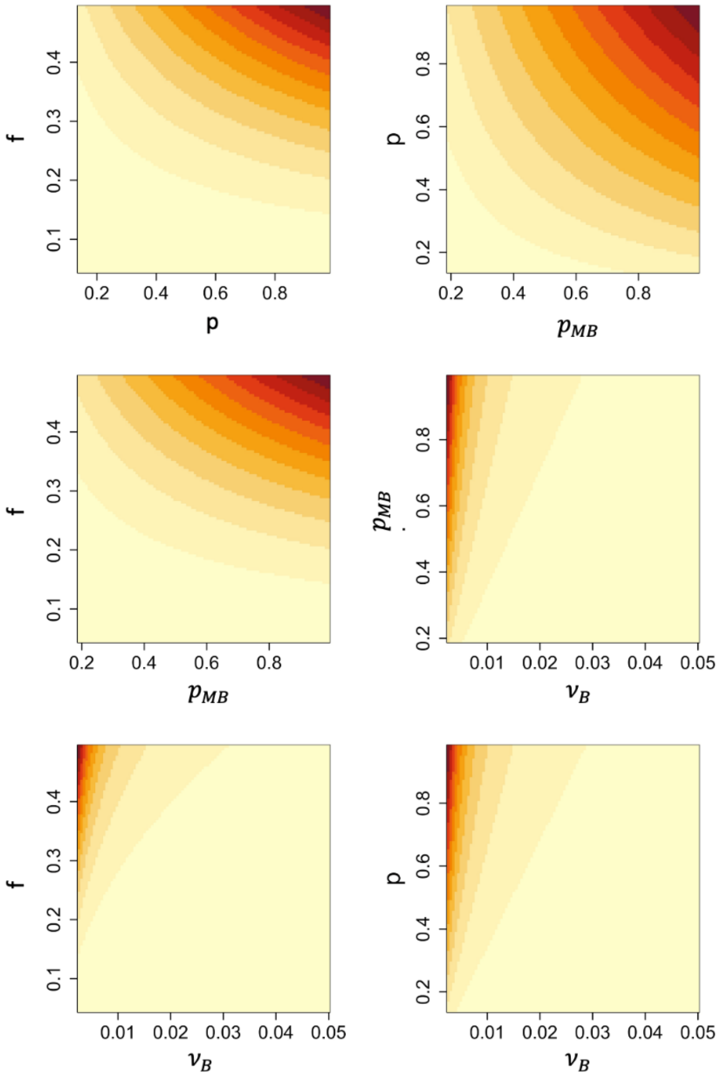

Figure H: Pairwise perturbation of epidemiological parameters.

References

1. Ciota AT, Matacchiero AC, Kilpatrick AM, Kramer LD. The Effect of Temperature on Life History Traits of Culex Mosquitoes. J Med Entomol. 2014;51: 55–62. doi:10.1603/ME13003
2. Marini G, Poletti P, Giacobini M, Pugliese A, Merler S, Rosà R. The Role of Climatic and Density Dependent Factors in Shaping Mosquito Population Dynamics: The Case of Culex pipiens in Northwestern Italy. PLOS ONE. 2016;11: e0154018. doi:10.1371/journal.pone.0154018
3. Vogels CBF, Fros JJ, Göertz GP, Pijlman GP, Koenraadt CJM. Vector competence of northern European Culex pipiens biotypes and hybrids for West Nile virus is differentially affected by temperature. Parasit Vectors. 2016;9: 393. doi:10.1186/s13071-016-1677-0
4. Reisen WK, Fang Y, Martinez VM. Effects of Temperature on the Transmission of West Nile Virus by Culex tarsalis (Diptera: Culicidae). J Med Entomol. 2006;43: 309–317. doi:10.1093/jmedent/43.2.309
5. Del Amo J, Llorente F, Pérez-Ramirez E, Soriguer RC, Figuerola J, Nowotny N, et al. Experimental infection of house sparrows (Passer domesticus) with West Nile virus strains of lineages 1 and 2. Vet Microbiol. 2014;172: 542–547. doi:10.1016/j.vetmic.2014.06.005
6. Summers-Smith JD. The sparrows: a study of the genus. Passer T AD Poyser Staff Engl. 1988.
7. Ewing DA, Cobbold CA, Purse BV, Nunn MA, White SM. Modelling the effect of temperature on the seasonal population dynamics of temperate mosquitoes. J Theor Biol. 2016;400: 65–79. doi:10.1016/j.jtbi.2016.04.008
8. Rizzoli A, Bolzoni L, Chadwick EA, Capelli G, Montarsi F, Grisenti M, et al. Understanding West Nile virus ecology in Europe: Culex pipiens host feeding preference in a hotspot of virus emergence. Parasit Vectors. 2015;8: 213. doi:10.1186/s13071-015-0831-4
