# Supplementary material for: Understanding West Nile virus transmission: Mathematical modelling to quantify the most critical parameters to predict infection dynamics
Source: PLoS Negl Trop Dis. 2023 May 1;17(5):e0010252. doi: 10.1371/journal.pntd.0010252 (PMC10174579; doi:10.1371/journal.pntd.0010252)
Supplement: Attachment — Submitted filename: Fesce et al_Response to reviewers.docx [file pntd.0010252.s005.docx]

**"Understanding West Nile virus spread: Mathematical modelling to determine the mechanisms with the most influence on infection dynamics."**

Dear Mrs Fesce,
 
Thank you very much for submitting your manuscript "Understanding West Nile virus spread: Mathematical modelling to determine the mechanisms with the most influence on infection dynamics." for consideration at PLOS Neglected Tropical Diseases. As with all papers reviewed by the journal, your manuscript was reviewed by members of the editorial board and by several independent reviewers. In light of the reviews (below this email), we would like to invite the resubmission of a significantly-revised version that takes into account the reviewers' comments.
 
The Authors are expected to address all the criticisms by all Reviewers. In particular, please provide more detailed description of the MCMC procedures, including convergence, autocorrelation analysis and thinning (Reviewers #1 & #2), consider the potential dependence between the 4 parameters of investigation (Reviewer #1), provide further justification for the selection of the 4 key parameters for assessment, reconsider the connection of the range for parameter assessment to the realistic parameter range (Reviewer #2) and clarify how Rt was derived and connected to time-dependent parameters (Reviewer #3). In additional to the above comments, please address,

1. What are the joint impact of the 4 key parameters? could some of the parameters may have synergistic effect on transmission? Some assessment (at least pairwise) would provide more insights from the study.

 
We cannot make any decision about publication until we have seen the revised manuscript and your response to the reviewers' comments. Your revised manuscript is also likely to be sent to reviewers for further evaluation.
 
When you are ready to resubmit, please upload the following:

[1] A letter containing a detailed list of your responses to the review comments and a description of the changes you have made in the manuscript. Please note while forming your response, if your article is accepted, you may have the opportunity to make the peer review history publicly available. The record will include editor decision letters (with reviews) and your responses to reviewer comments. If eligible, we will contact you to opt in or out.

[2] Two versions of the revised manuscript: one with either highlights or tracked changes denoting where the text has been changed; the other a clean version (uploaded as the manuscript file).

Important additional instructions are given below your reviewer comments.

Please prepare and submit your revised manuscript within 60 days. If you anticipate any delay, please let us know the expected resubmission date by replying to this email. Please note that revised manuscripts received after the 60-day due date may require evaluation and peer review similar to newly submitted manuscripts.
 
Thank you again for your submission. We hope that our editorial process has been constructive so far, and we welcome your feedback at any time. Please don't hesitate to contact us if you have any questions or comments.
 
Sincerely,

Eric HY Lau, Ph.D.
Associate Editor
PLOS Neglected Tropical Diseases

Benjamin Althouse
Deputy Editor
PLOS Neglected Tropical Diseases

***********************

**We thank the Editor and all the reviewers for their evaluation of the manuscript and all their constructive comments and criticisms. Given the existing knowledge gaps about West Nile disease transmission processes, by writing the present work we wanted to highlight where the main gaps are. Our aim is to provide a quantitative estimate of the impact of different parameters in the prediction of West Nile spread, thus providing a baseline to direct future investigations. For this reason, in the first version especially, we wanted to provide an application of mathematical modelling aimed at being accessible to a broad audience of scientists, not modellers only. We are thankful to the reviewers that spotted the weaknesses of the ms, especially those regarding the more technical mathematical aspects, giving us the opportunity to improve our work. According with reviewer’s suggestion, we modified the title of this ms, that we believe now better matches with its aim. To meet reviewer’s requests, we performed the analyses using a new set of parameters. As a consequence, we revised the visualization and details of our results. We also included all over the text more details about the methods used, we explained more in depth the reason of our choice about which parameters to investigate and we reconsidered the ranges for parameter for the sensitivity analysis. Furthermore, we provide more details about how these parameters were estimated and about interdependencies between them.**

**Thanks to Editor’s and Reviewer’s suggestions, we are confident that this second version has highly improved in robustness and readability. We also sincerely hope to have adequately addressed all the points raised by the reviewers to fit PLOS Neglected Tropical Diseases required standards**

**On behalf of all the Authors**

**Sincerely,**

**Elisa Fesce**

The Authors are expected to address all the criticisms by all Reviewers. In particular, please provide more detailed description of the MCMC procedures, including convergence, autocorrelation analysis and thinning (Reviewers #1 & #2), consider the potential dependence between the 4 parameters of investigation (Reviewer #1), provide further justification for the selection of the 4 key parameters for assessment, reconsider the connection of the range for parameter assessment to the realistic parameter range (Reviewer #2) and clarify how Rt was derived and connected to time-dependent parameters (Reviewer #3). In additional to the above comments, please address,

1. What are the joint impact of the 4 key parameters? could some of the parameters may have synergistic effect on transmission? Some assessment (at least pairwise) would provide more insights from the study.

**R: We thank the Editor and all the reviewers for the comments and criticisms, which we addressed in the current version of the manuscript and in the following document. In addition, to address the request of investigating the joint impact of the 4 key parameters, we added in the supplementary materials their pairwise perturbation analysis, showing no synergistic effect.**

Reviewer's Responses to Questions

**Key Review Criteria Required for Acceptance?**


As you describe the new analyses required for acceptance, please consider the following:


**Methods**


-Are the objectives of the study clearly articulated with a clear testable hypothesis stated?

-Is the study design appropriate to address the stated objectives?

-Is the population clearly described and appropriate for the hypothesis being tested?

-Is the sample size sufficient to ensure adequate power to address the hypothesis being tested?

-Were correct statistical analysis used to support conclusions?

-Are there concerns about ethical or regulatory requirements being met?


Reviewer #1: (No Response)

Reviewer #2: (No Response)

Reviewer #3: 2 - The authors should clearly stated throughout the paper that this study just deal with single mosquito species and, most importantly, single bird species.

3 - R_t estimate: the rational and derivation are not clear and need to be explained. Some included parameters are supposed to change over time and it is hardly understandable that the final expression of R_r looks very similar to R_0

4 - Provide a clear definition of the spread probability and how is this computed. In addition, I would like to remind to the authors that the probability of infection spread is not "1" even for R_0 >1.

--------------------

**Results**


-Does the analysis presented match the analysis plan?

-Are the results clearly and completely presented?

-Are the figures (Tables, Images) of sufficient quality for clarity?


Reviewer #1: (No Response)

Reviewer #2: (No Response)

Reviewer #3: 5 - Figues in the Appendix are missing

6 - S1 Appendix, Table A: Something missing in the expression or value of Theta_M

7 - Figure quality should be improved

--------------------

**Conclusions**


-Are the conclusions supported by the data presented?

-Are the limitations of analysis clearly described?

-Do the authors discuss how these data can be helpful to advance our understanding of the topic under study?

-Is public health relevance addressed?


Reviewer #1: (No Response)

Reviewer #2: (No Response)

Reviewer #3: (No Response)

--------------------

**Editorial and Data Presentation Modifications?**


Use this section for editorial suggestions as well as relatively minor modifications of existing data that would enhance clarity. If the only modifications needed are minor and/or editorial, you may wish to recommend “Minor Revision” or “Accept”.

Reviewer #1: (No Response)

Reviewer #2: (No Response)

Reviewer #3: (No Response)

--------------------

**Summary and General Comments**

**Reviewer #1:**

In this manuscript titled “Understanding West Nile virus spread: Mathematical modelling to determine the mechanisms with the most influence on infection dynamics”, the authors investigate the mechanisms most affecting the transmission of Culex pipiens-borne West Nile disease by analysing a mathematical model of vector and transmission dynamics. The authors modify the model published in Marini et al. (2020), incorporate recently collected field data, and perform sensitivity analysis on a subset of parameters relevant to disease transmission.

Several vector species, their respective biting preferences, and reservoir species are involved in sustaining and spreading the virus. Several approaches for modelling the underlying mechanisms have been proposed (by the same group and others such as Bergsman et al. 2016). The authors present a technical analysis of a particular model, but not sufficiently novel evidence to improve the parameterisation of the model or the understanding of the disease mechanism. One might wonder if the authors would obtain the same conclusions, had they used a different model. Overall, in its current state, the manuscript seems appropriate for a more technical audience.

**R: We thank the reviewer for all the constructive comments and for acknowledging the relevance of the work for a technical audience.**

**In particular, we agree that the choice of different models and/or assumptions can eventually lead to a different estimate of the R_0_ (and R_t_). Most of the literature agrees to consider West Nile disease well described by a SEIR/SIR model for the infection in birds and a SEI/SI model for the infection in mosquitoes (Wonham et al 2004, Bergsman et al. 2016, Marini et al 2018). This is the main reason that drove us to not explore different mechanisms/routes of transmission. Furthermore, we chose to base our simulation on a model that has already been validated in Italy, to guarantee further robustness of the results. We do acknowledge that our choice is not the only possible one, and it is possible to further investigate the system through a different model. We added lines 512-514 in the discussion to acknowledge for this limitation.**

**With the present work we aimed to explore the impact of parameter estimates on our ability to predict WNV spread. We then better stated the aims of this work (lines 99-100 in the introduction and lines 380-387 in the discussion), furthermore we replaced “transmission mechanisms” with “transmission processes” throughout the ms.**

The authors adapted a recently published model to perform the analysis, giving more emphasis to the mosquito-bird interactions. However, it is not clear in the text why this adaptation is necessary, and why the analysis could not be performed on the original model. It is also not clear how the model was adapted. An extended description of the changes would add significantly to the clarity of the manuscript.

**R: To address this comment, we added lines 93-98 in the introduction and lines 387-389 in the discussion to highlight why it can be worth to adapt a model to a new dataset. Furthermore, in “Model Structure” paragraph (lines 141-144) we better explained that the main change we did to the structure of the model was to include another parameter representing the competence of the avian species. This change was made in order to include the possibility that different avian species could develop different viraemic level, after a mosquito bite.**

A major issue in the analysis is the authors’ statement of performing 10,000 iterations of MCMC to sample 10,000 parameter sets from the posterior distribution, which implies that the initial transient state of the MCMC chain was not discarded, autocorrelation was not checked, and thinning (where every n-th iteration is selected to prevent autocorrelation) was not applied. Although the authors state that a random sub-sample of 100 parameters sets were drawn from this reservoir set, neither these sub-samples nor the original sample can be regarded as proper samples from the posterior distribution without the validation steps mentioned above.

**R: We thank the reviewer for the comment. In order to address these issues, and provide a better description of methods, we performed all analysis applying thinning and re-sampling the 100 sets of parameters used for the simulations. To lower autocorrelation the number of iterations of the MCMC was brought from 10,000 to 100,000 (line 210). We updated lines 199-215 in the methods to include these changes and to provide a clearer description of the methods used. Even if the simulations with the new set of parameters did not substantially change the meaning of our results, we however adapted the results presentation and visualisation, and the relative discussion.**

The authors performed the sensitivity analysis on 4 parameters. Although the choice of these are justified, due to the uncertainty in the other parameters (due to experimental errors or uncertainties in inference), a broader analysis including other relevant parameters might also be helpful. More importantly, the analysis was performed on each parameter independently, however, their potential interdependencies necessitate investigating the impact of pairwise or random perturbations on all parameters.

**R: We thank the reviewer for the suggestion. We decided to focus on these four parameters because these are the main parameters related to the avian community that could be further investigated by field or laboratory experiments. Our choice of focusing on the avian community relies on the following reasons: there is a wide variety of avian species; their presence, abundance and distribution are peculiar of each area; and experimentation on birds is more demanding and can rely on lower numbers compared to the one on mosquitoes. We added lines 105-113 in the introduction and lines 381-387 in the discussion to provide a better explanation of our choice. Furthermore, to provide a clearer description of the parameter related to the mosquito biting rate, we modified lines 182-188. To avoid confusion between biting rate and fraction of bites, we changed the name of the parameters representing the fraction of bites directed to competent birds from b1 and b2 to f1 and f2. As no correlation was found among epidemiological parameters, for the sake of simplicity, we only reported the sensitivity analysis performed on each parameter independently. Despite that a visual inspection of pairwise perturbations was performed, showing no combined effect of parameter variation (lines 346-348 and supplementary Fig H).**

Minor points:
- The statement in line 440 should not be interpreted in the biological context. A few initial infectious mosquitoes and a low number of birds might be enough to start and maintain the infection at the beginning of the season in the context of this deterministic model, but hardly ever any biological conclusions can be drawn from this. When geospatial range and stochastic interactions dominate, the probability of sustaining the infection may decrease considerably.

**R: We thank the reviewer for the observation, we acknowledged in the discussion (line 500) that our model has the limitation of not considering stochasticity being a deterministic model**

- The Supplementary Figure S1 detailing the goodness of fit could have been more informative had it resolved the values less than 5, where most of the data seems to be.

**R: According with reviewer’s suggestion, we modified Fig B in Supplementary Materials by restricting from 0 to 5 the y-axis.**

- Having a different order of the same parameters in Figures 4 and 5 is confusing.

**R: We thank the reviewer for the observation, there was a typo in the order of parameters of figure 4, in the updated version of figures parameters have the same order in all figures.**

- I noticed a typo in S1 Appendix Table A, the mosquito death rate parameter 4.75 was originally given as 4.57 in Marini et al. (2020).

**R: We thank the reviewer for the observation, we corrected the typo.**

**Reviewer #2:**

The authors present a sensitivity analysis of an existing mathematical model to identify the parameters which are the most influential in determining the risk of West Nile virus transmission. The study also estimates these parameters based on field data collected in Northern Italy. The authors are certainly correct that the parameters they study are poorly understood, particularly in a European context, and so highlighting the importance of these parameters given the results of the mathematical model is an important and worthwhile endeavour. It must be noted that many of the results highlighted may already be known to those familiar with R0 (or time-varying R0) equations for vector-borne diseases and their interpretation (for example, biting rate is relatively important because it appears as a squared term in the R0 equation where the terms governing transmission probabilities are not squared); however, as this may be a relatively small subset of the readership of the journal the paper is still likely to be of reasonably board interest and appeal. Further, the estimates of these parameters based on the field data (whilst very variable in some cases) will be of wide interest and may benefit future modelling studies. Nonetheless, I do have some concerns about some of the analyses and presentation which I feel need addressed prior to publication. Full details of my concerns and suggestions are given below:

**R: We thank the Reviewer for all the constructive specific comments, and, in this general comment, for acknowledging the relevance of our analyses and their practical implications in supporting future investigations. We agree that some of the results may be inferred by an analytical analysis of the R_0_. Despite that, as was already accounted by the reviewer, we aimed at targeting our analysis to a broader range of scientists and field workers, which might or might not be satisfied by a mere theoretical demonstration of results. We firmly believe in the potential of applying mathematical modelling to address base and field research, but we do also think that is very important for modellers to show results in a clear and immediate way to make them fully available to the whole scientific community.**

- I would suggest that a more thorough justification for the 4 parameters chosen for further study is needed, as this choice is clearly central to the paper and very important. At present there is little justification beyond the authors stating they are the most important. Whilst I personally mostly agree, I still think more explanation should be given (why not host to host transmission or vertical transmission, for example?). In particular, I would question why the duration of the incubation period of the virus within the mosquito was not included as, to my knowledge, there is substantial uncertainty surrounding this parameter?

**R: We thank the reviewer for highlighting this point. Due to model structure and the already high number of unknown parameters, we thought that including much more complexity could have been counteractive. Indeed, for most of the Italian/European avian species, there are very few information about the epidemiological parameter needed to model WNV dynamics. This is the reason why we opted for a simpler model that does not include different routes of transmission (e.g. host-to-host or vertical transmission). We indeed believe that, given all present knowledge gaps, adding further complexity to the model would inflate the uncertainty of results, depriving our analysis of the informative indications. For the same reason, as the major knowledge gaps about epidemiological parameters are related to the bird species, and at the same time field and laboratory experiments are more complex and demanding to perform, we decided to focus on these four parameters. We added lines 178-179 in the method session and lines 500-504 acknowledging for those limitations. We also added lines 105-113 in the introduction and lines 381-387 in the discussion to further justify our choices about what parameters to include in the present analysis. Furthermore, we provided a clearer description of the parameter related to the mosquito biting rate in lined 181-188. To avoid confusion between biting rate (b, that is a function of fraction of bites and temperature) and the fraction of bites, we changed the parameter name from b1 and b2 to f1 and f2 (representing the fraction of bites).**

- The authors choose the parameters p (avian competence to infection) and p_MB (avian susceptibility to infection) for inclusion in their analysis and the analyses then centre around the calculation of Rt (lines 246-249) and understanding how percentage changes in the parameters will affect Rt. It can be seen from the equation that both of these parameters (p and p_MB) will have a linear effect on Rt (e.g. a 50% decrease in either parameter will cause a 50% decrease in Rt). This is indeed what the authors go on to show in Figure 4 but it begs the question of why to include both parameters when they are essentially identical within the model (albeit with a small difference when calculating spread probability due to the thresholding of Rt at 1)?

**R: We further thank the reviewer for this comment. We do agree that it is possible to see from the equation that both of the parameters have a linear effect on R_0_ and Rt. Despite that, the two parameters have a very different biological meaning and lead to very different types of experiments to be further investigated. For this reason, and our will to reach a wider spectrum of researchers, we decided to include them both in our analysis and we acknowledged these aspects in lines 156-158and 404-405.**

- Related to the above point, both of the parameters p and p_MB are defined as probabilities with baseline values of 0.62 and 0.78 respectively (lines 204-207 and Table 1), so a 90% increase in these parameters would lead to a probability greater than 1. I had initially assumed that the values would be capped to 1, however the sustained linear increase in Figure 4 suggests not. Am I misunderstanding something or are there probabilities >1?

**R: We thank the reviewer for pointing this out, as following the next comments we investigated changes at fixed centiles across the range of the 95% of the confidence interval from the MCMC, the maximum values explored by the sensitivity analysis for p and pMB are respectively 0.98 and 0.99.**

- I worry that there is a bit of a problem with the approach of considering percentage changes for each of the parameters. A lot of what comes out as important here depends on the model structure. For example, the authors have identified the bird recovery rate as very important, however much of this stems from the fact that it appears in the denominator of the Rt equation and so as the recovery rate approaches 0 Rt will approach infinity. Thus, if we consider a 90% reduction from the baseline then the recovery rate gets close to zero (it takes nearly 4 weeks to recover) and we see a much bigger increase in Rt than for the other parameters which appear in the numerator; however, if we consider a 90% increase from the baseline estimates then recovery rate actually results in the smallest difference of the 4 parameters but this difference is less noticeable when plotted.

**R: As this and the following comment relate to the same issue, we addressed them both by changing parameter values ranges used for the sensitivity analysis. A full description of the changes is then provided as a response to the next comment.**

This 90% decrease in the recovery rate results in a value beyond the lower end of the 95% interval stemming from the MCMC, which begs the question of whether or not this is really the most important parameter if the biggest effect on Rt comes when we reach values which appear highly unlikely given the field data? Why not investigate changes at fixed centiles across the range of the 95% interval from the MCMC? This way the changes for each parameter are across the range of the most plausible values for each parameter? The results might be similar but I feel there is some nuance being missed here whereby, for example, the infectious period in birds may only be particularly influential if the true value is in fact very low (and possibly lower than the field data would suggest).

**R: We thank the reviewer for this comment and suggestion. Accordingly, we estimated and visualised our results by changing parameter values across a range of our estimates from the MCMC (lines 297-300 in the methods session and results between lines 339-378). For the sake of simplicity, we decided to perform the analysis on the R0, thus reducing variability due to differences in the number of susceptible birds across the season (lines 300-302). Furthermore, to be more realistic and account for the existing differences in vector-host ratios across subareas and years, we showed the sensitivity in each subarea and year (different lines in figure 4). For a more complete interpretation of results, we also added a grey boxplot representing the posterior distributions of parameters. We consequently adapted our description and discussion of results.**

- I see that the authors have addressed some of the concerns in my previous point in the discussion where they highlight that the bird recovery rate is only particularly influential if it is in fact very low. This discussion was very welcome and I feel that this caveat should be made more apparent elsewhere. For example, at the start and end of the discussion the authors state that the bird recovery rate is very important without stating this caveat and I feel it should be included in those places.

**R: We thank the reviewer for the kind comment, we acknowledged for it in lines 390-393 in the discussion.**

More minor comments and suggestions:
- Line 67 – “approximately 25%”
- Lines 70-71 – say when WNV was introduced for the first time?
- Line 76 – delete encountered
- Line 82 – “potentially inaccurate” since we don’t actually know how inaccurate (if at all) they will be
- Lines 90-93 – maybe add a reference?

**R: We thank the reviewer for the very appropriate minor comments, we addressed them all in the ms.**

- Daily mean temperatures have been used throughout. Given what we know about the importance of diurnal temperature variation I wondered why that was not incorporated?

**R: Given the complexity of the system, the high number of unknowns already included in the model, and the aim of the work to investigate the effect of a change in parameter estimates, we considered the effect of diurnal temperature variation negligible. Furthermore, we chose to incorporate only the mean daily temperature because the model timestep was considered one day and all model parameters are estimated on a daily base. Despite thus going beyond the scope of our work, the comment is a good suggestion for further developments of the model proposed.**

- I think there should be some more details about the MCMC procedure given. For example, say something about checking for convergence and/or autocorrelation in the chains? I'd also like to know how the priors were chosen - I couldn't find this here or in a brief scan of the previous paper.

**R: To address this and a previous comment of reviewer #1, we provided a more detailed description of the methods used in lines 199-213. We added some plots of the autocorrelation of parameters in the supplementary materials and we stated that we check for convergence via visual inspection in lines 213-215. Furthermore, we added a column in Table 1 in the main text showing the prior distributions chosen.**

- The assumption that birds do not lose immunity seems likely to be a big and potentially influential one. It would be good if there was a reference to support birds maintaining immunity for a long time. If there is no such reference because it has not been studied then would it not be important to consider a further scenario where birds lost their immunity?

**R: The role of bird immunity in WNV spread is indeed very important and debated point. We decided not to take it into account in the present work as, given the importance of a further scenario where birds lost their immunity, we aimed to investigate this aspect in a future work supported by specific analyses and by field experiment data. We added lines 502-504 to account for this limitation of the ms proposed.**

- Lines 231 and 249 – what is delta_B here? I can’t find it defined. I’m assuming this is a typo and it should be the bird recovery rate, nu_B.

**R: We thank the reviewer for pointing out the typo, that has now been corrected.**

- Lines 299-302 - Suggest rewording to something like "No meaningful correlation" considering you filter on both p-value and correlation strength. If, for example, r=0.3 and p=0.01 then there is reasonable evidence there might be a correlation, just one that is weak enough that it may not be interesting. Also, why are the last two of the three correlations mentioned included? They have tiny correlation values and high p-values and don't meet the criteria for a correlation specified above.

**R: We accepted reviewer suggestions by modifying line 329 in “no meaningful correlation”. Furthermore, as the posterior distributions of parameters have been modified according with previous comments, we updated the analysis of correlation among parameters and lines 329-332 consequently.**

- Lines 309 – 320 and Fig 4 – The figure labelling has gone wrong here. The (A)-(D) in the text don’t match up with the Figure.
- Line 328 – “the least” rather than “a less”
- Line 330 – The authors highlight August/September here but I think May looks similar.
- Line 331 – I think August and May look just as variable as July and September.

**R: We thank the reviewer for the minor corrections that have been addressed in the ms.**

- Line 333 – “showed similar results” – Given the two parameters have the same effect on Rt then I think these results will be identical, no?
- Lines 333-334 - I think the authors mean increased here because the first half of the sentence talks about a decrease. If we are considering the increase then the effect in p will be slightly larger than p_MB because the baseline value of p is slightly lower and as these are probabilities there is a threshold on how much they can be increased. So I think this may be the only case where I would expect that there may indeed be a small difference between the two parameters (unless I’m fundamentally misunderstanding something). I would still like some confirmation that these probabilities are not being allowed to become greater than 1 though.

**R: We thank the reviewer for pointing this out. By modifying the ranges for the changes of parameter values as previously suggested and described, we overcame the possibility of p and pBM to be greater than 1. It follows that the differences reported in the effects of the two parameters are due to the differences in the ranges of the 95% interval from the MCMC among the two parameters.**

- Line 343 – I find this sentence confusing. I’m not sure what the authors are referring to when they say “lower than that for”. What is “that” in this context?

**R: We changed the sentence in “In accordance with previous analyses ,for all tested vector-host ratios, Rt was always equal to zero for any tested temperature below 14 °C”, now at line 371-372 to keep it more clear.**

- Lines 439-441 – I’m struggling to remember where this was shown. Include a reference to the relevant Figure or Table.

**R: We thank the reviewer for this comment, we changed the sentence (now at line 488-489) to make it more coherent with shown results.**

- There's very little known about these parameters in Europe but there is a bit more in North America. It might be interesting to add a paragraph in the discussion comparing the values here to some of the values there? This is very much just a suggestion though, if the author's disagree then I am happy to be ignored.

**R: We thank the reviewer for this suggestion, we acknowledge this comment by mentioning the existence of better estimates for these parameters for American bird species in lines 504-512, where we also further explain or choice of investigating those four parameters. The suggestion of the reviewer is very interesting, and it would be great to have the possibility to compare values obtained from European species to those available for American species. Some comparisons are available for magpies and house sparrows, and partially motivated our study. Despite that, given the theoretical approach here adopted, some of the model limitations (like accounting for only one competent species) and the very wide confidence intervals that we obtained for parameter estimates, we believe that the comparison suggested goes beyond the scopes and possibilities of the present work.**

**Reviewer #3:**

This paper deals with the understanding of the spread of West Nile Virus (WNV) in Lombardi, northern Italy. The authors propose a sensitivity analysis to identify the parameters with the largest impact on the spread of the WNV infection. They used a mathematical model simulating WNV spread to estimate the basic reproduction number of the infection and to quantify the infection spread in mosquitoes (Culux pipes only) and birds (magpies). The focus was on how the following four parameters might affect WNV transmission: i) mosquito biting rate on birds, ii) avian competence, iii) the duration of the infectious period in birds or avian recovery and iv) avian susceptibility to WNV infection. They found that the duration of the infectious period in birds and mosquito biting rate are the most impactful parameters.

Although all of this is not new at all, the paper is well written and report interesting results that are worthwhile to be published. Therefore, I would recommend to publish this material in PNTD after the authors have considered the following revisions.

**R: We kindly thank the reviewer for acknowledging the relevance of the work proposed an for all the constructive comments.**

1 - Title: There is no new mechanism of WNV transmission. The authors rather carried out an estimation of some parameters of the problem. Revise the title to reflect that.

**R: We embraced reviewer’s comment by rephrasing the title as follows:**

**“Understanding West Nile virus transmission: mathematical modelling to quantify the most critical parameters to predict infection dynamics”**

2 - The authors should clearly stated throughout the paper that this study just deal with single mosquito species and, most importantly, single bird species.

**R: We do agree with the reviewer that this aspect of the proposed work needs to be clearly stated. For this reason, we modified line 148 (in the methods) to state more clearly that we are modelling one single bird species.**

3 - R_t estimate: the rational and derivation are not clear and need to be explained. Some included parameters are supposed to change over time and it is hardly understandable that the final expression of R_r looks very similar to R_0

**R: We thank the reviewer for stating this out, we modified lines 263-268, 279, 300-302 and 303-305to provide a better explanation of the formula used for estimating R0 and Rt.**

4 - Provide a clear definition of the spread probability and how is this computed. In addition, I would like to remind to the authors that the probability of infection spread is not "1" even for R_0 >1.

**R: We thank the reviewer for the comment, we modified lines 287-289 and we changed throughout the ms the caption “spread probability” with “outbreak frequency to account for it.**

5 - Figues in the Appendix are missing

**R: We feel sorry for the inconvenience, we checked that figures are present and meet the requirements of PLOS Neglected Tropical Diseases.**

6 - S1 Appendix, Table A: Something missing in the expression or value of Theta_M

**R: We thank the reviewer for pointing out the typo, that was corrected.**

7 - Figure quality should be improved

**R: We thank the reviewer for the suggestion, we improved figures quality and checked they meet PLOS Neglected Tropical Diseases requirements.**
