# Supplementary material for: Understanding West Nile virus transmission: Mathematical modelling to quantify the most critical parameters to predict infection dynamics
Source: PLoS Negl Trop Dis. 2023 May 1;17(5):e0010252. doi: 10.1371/journal.pntd.0010252 (PMC10174579; doi:10.1371/journal.pntd.0010252)
Supplement: Attachment — Submitted filename: Fesce et al_Response to reviewers.docx [file pntd.0010252.s006.docx]

01/12/22

Dear Mrs Fesce,
 
Thank you very much for submitting your manuscript "Understanding West Nile virus transmission: mathematical modelling to quantify the most critical parameters to predict infection dynamics" for consideration at PLOS Neglected Tropical Diseases. As with all papers reviewed by the journal, your manuscript was reviewed by members of the editorial board and by several independent reviewers. In light of the reviews (below this email), we would like to invite the resubmission of a significantly-revised version that takes into account the reviewers' comments.
 
The Authors are expected to address all the criticisms by all Reviewers. In particular, please confirm the interpretation of the parameters, and the associations between parameters have to be considered in the analyses and interpretation (Reviewer #1). In additional to the above comments, please address,

1.Abstract, “This analysis allows us to identify the investigated parameters that need the most accurate estimate and to further investigate transmission processes that majorly affect WNV spread.” In Table 1, the estimated recovery rate had a very wide 95% CI (0.002-0.94). Most of the parameters had very wide 95% CI too almost covering >80% of the plausible parameter range. Please take this into account and revise the abstract/discussion.
2. Table 1, all the credible intervals for the estimated parameters covered almost the entire range of the prior distributions. This is probably an indication that the model and data could not provide reliable estimates for any of the parameters. One possible improvement is to use more information priors based on the literature. This is important as quantification of parameters is a main study objective but this is not yet achieved.
3. Table 2 shows the upper bound of some parameters would lead to implausible R0 (>1000). This is probably an issue related to the associations between parameters and hence parameters should not be analyzed independently.

 
We cannot make any decision about publication until we have seen the revised manuscript and your response to the reviewers' comments. Your revised manuscript is also likely to be sent to reviewers for further evaluation.
 
When you are ready to resubmit, please upload the following:

[1] A letter containing a detailed list of your responses to the review comments and a description of the changes you have made in the manuscript. Please note while forming your response, if your article is accepted, you may have the opportunity to make the peer review history publicly available. The record will include editor decision letters (with reviews) and your responses to reviewer comments. If eligible, we will contact you to opt in or out.

[2] Two versions of the revised manuscript: one with either highlights or tracked changes denoting where the text has been changed; the other a clean version (uploaded as the manuscript file).

Important additional instructions are given below your reviewer comments.

Please prepare and submit your revised manuscript within 60 days. If you anticipate any delay, please let us know the expected resubmission date by replying to this email. Please note that revised manuscripts received after the 60-day due date may require evaluation and peer review similar to newly submitted manuscripts.
 
Thank you again for your submission. We hope that our editorial process has been constructive so far, and we welcome your feedback at any time. Please don't hesitate to contact us if you have any questions or comments.
 
Sincerely,

Eric HY Lau, Ph.D.
Academic Editor
PLOS Neglected Tropical Diseases

Benjamin Althouse
Section Editor
PLOS Neglected Tropical Diseases

***********************

The Authors are expected to address all the criticisms by all Reviewers. In particular, please confirm the interpretation of the parameters, and the associations between parameters have to be considered in the analyses and interpretation (Reviewer #1). In additional to the above comments, please address,

1. Abstract, “This analysis allows us to identify the investigated parameters that need the most accurate estimate and to further investigate transmission processes that majorly affect WNV spread.” In Table 1, the estimated recovery rate had a very wide 95% CI (0.002-0.94). Most of the parameters had very wide 95% CI too almost covering >80% of the plausible parameter range. Please take this into account and revise the abstract/discussion.

***R: We thank the editor that by highlighting this point gave us the opportunity to better state the limits and objectives of the proposed model. We edited the abstract and the discussion following the editor’s & Reviewer’s comment. We edited lines 30-31 to explicitly state that several knowledge gaps are still unfilled in WNV epidemiology. We also clarified that our work does not aim at filling all of them, but aims at disentangling what investigation could be more efficient in improving our knowledge of WNV epidemiology (lines 34-35 and lines 40-44). We also edited the sentence “This analysis allows us to identify the investigated parameters that need the most accurate estimate and to further investigate transmission processes that majorly affect WNV spread.” in the author summary (lines 58-60) according with editor’s suggestions. We also edited lines 34-35, 382 and 386-387 to acknowledge for our choice in investigating those four parameters in particular. Furthermore, in the Discussion we edited lines 388-389 to highlight that the conclusions we drawn comes from the sensitivity analysis performed, and we also added lines 533-539 to acknowledge for the wide CI we found, and further state that this work alone is not enough to fill all the gaps we still have, but only to pinpoint where it would be more efficient to address future studies.***

2. Table 1, all the credible intervals for the estimated parameters covered almost the entire range of the prior distributions. This is probably an indication that the model and data could not provide reliable estimates for any of the parameters. One possible improvement is to use more information priors based on the literature. This is important as quantification of parameters is a main study objective but this is not yet achieved.

***R: We thank the editor for the criticism and suggestion, we added lines 533-539 to fully discuss this and the previous point. In detail in those lines we clarified that we chose to keep the ranges of the priors of parameters the widest possible, forsaking some precision in model fit, not to risk to influence our analysis with possibly biased pre-existing information. We indeed believe that the site-specificity of the composition of avian community might affect parameter estimates. As scarce information is available on avian communities (particularly for our study area), we believe that a mere transposition of assumptions from other areas can be misleading. For that reason, we decided to keep a wide uncertainty in parameter prior distributions and evaluate the effect of a parameter change without restricting their range of existence.***

3. Table 2 shows the upper bound of some parameters would lead to implausible R0 (>1000). This is probably an issue related to the associations between parameters and hence parameters should not be analyzed independently.

***R: We thank the editor for pointing this out. We acknowledged the possibility of an over-estimate of Ro/Rt (that can be easily caused by an estimate of the recovery rate close to zero) in the discussion (lines 539-543).***

Reviewer's Responses to Questions

**Key Review Criteria Required for Acceptance?**


As you describe the new analyses required for acceptance, please consider the following:


**Methods**


-Are the objectives of the study clearly articulated with a clear testable hypothesis stated?

-Is the study design appropriate to address the stated objectives?

-Is the population clearly described and appropriate for the hypothesis being tested?

-Is the sample size sufficient to ensure adequate power to address the hypothesis being tested?

-Were correct statistical analysis used to support conclusions?

-Are there concerns about ethical or regulatory requirements being met?


Reviewer #1: (No Response)

Reviewer #2: (No Response)

Reviewer #3: (No Response)

--------------------

**Results**


-Does the analysis presented match the analysis plan?

-Are the results clearly and completely presented?

-Are the figures (Tables, Images) of sufficient quality for clarity?


Reviewer #1: (No Response)

Reviewer #2: (No Response)

Reviewer #3: (No Response)

--------------------

**Conclusions**


-Are the conclusions supported by the data presented?

-Are the limitations of analysis clearly described?

-Do the authors discuss how these data can be helpful to advance our understanding of the topic under study?

-Is public health relevance addressed?


Reviewer #1: (No Response)

Reviewer #2: (No Response)

Reviewer #3: (No Response)

--------------------

**Editorial and Data Presentation Modifications?**


Use this section for editorial suggestions as well as relatively minor modifications of existing data that would enhance clarity. If the only modifications needed are minor and/or editorial, you may wish to recommend “Minor Revision” or “Accept”.

Reviewer #1: (No Response)

Reviewer #2: (No Response)

Reviewer #3: (No Response)

--------------------

**Summary and General Comments**


Use this section to provide overall comments, discuss strengths/weaknesses of the study, novelty, significance, general execution and scholarship. You may also include additional comments for the author, including concerns about dual publication, research ethics, or publication ethics. If requesting major revision, please articulate the new experiments that are needed.

**Reviewer #1**

I thank the authors for their efforts in preparing this improved version of the manuscript. I am pleased to notice that most of my concerns have been adequately addressed. However, there still remains a couple of major issues to be addressed in further revision.

The authors changed the statement "Moreover, our model shows that few initial infectious mosquitoes and a low number of birds are enough to start and maintain the infection at the beginning of the season" into "Moreover, we showed that a low vector-host ratio (100 mosquitoes/host) and an initial number of competent hosts lower 5 (table 1) are enough to start and maintain the infection at the beginning of the season." and added "Also, the model provided is deterministic, thus not taking into account any stochasticity of events." later in the paragraph. It is not clear to me how the authors derive this conclusion about the impact of vector-host ratio and initial number of competent hosts on R0 at the beginning of the season. Nevertheless, these changes do not address my earlier concerns that a deterministic model is being interpreted erroneously.

***R: We thank the reviewer for highlighting this point. We edited lines 488-514 in the discussion to better explain our conclusions and to better acknowledge for the limits of the use of a deterministic model. Hoping to have correctly interpreted the criticism of the reviewer, we explained the line of reasoning that brought us to conclude that the vector host ratio has an impact on WNV spread. Furthermore, we acknowledged the caveat of the use of a deterministic model in the early stages of infection.***

Related to this issue, in line 289, 357, and in the Results section "Transmission maintenance during seasons", the frequency of simulations (obtained from the posterior distribution of parameter estimates) leading to R0 > 1 is used to indicate the frequency (or probability) of an outbreak. I believe the "outbreak frequency" referred by the authors is in fact an estimation of R0, and the range of R0 simulated using the posterior samples is the range in R0. In this case, interpreting the quantity as outbreak frequency (or probability) is misleading. I suggest that authors display the value of R0 and its variability, and assess risk based on the times when the average R0 is more than 1. This also applies to the interpretation of Rt in the final section of Results.

***R: If we have interpreted the comment correctly, the approach suggested is an alternative elegant way to show our results. Despite that, we decided to show the results as the “proportion of R0>1 over the number of simulations performed” due to the high variability of ranges of parameters we accounted for. We chose this approach because it shows if we are (or are not) likely to have WNV spread on a given month besides the parameter estimates.***

The authors claim that there are no associations among the transmission parameters. However, they have chosen to investigate a subset among all the inferred parameters. Evidently, these have no significant associations among them. The posterior samples clearly show, as the authors have also displayed in S1 Fig. F, there are significant associations among the inferred transmission parameters including the following: iBw and iBe are strongly positively correlated, and ai and nuB are strongly negatively correlated. Perhaps the authors mean "no association between the bird-related transmission parameters chosen for analysis in this context".

***R: We thank the reviewer for pointing out this typo in the supplementary materials, we edited it as suggested (lines 71-75 S1).***

Minor points
=========

In line 183, the reference cited is a book by Anderson titled "Biology of the Ubiquitous House Sparrow: From Genes to Populations". I am not able to confirm any reference to mosquito biting rate quantification in this book. However, I urge the authors to double-check if this is the intended citation.

Also, in lines 203-4, the proposed likelihood function does not appear in the cited manuscript #25 (Moschini et al. 2017).

***R: We thank the reviewer for spotting the typo, we amended the two references (now lines 184 and 204).***

In line 238, all prior distributions pertaining the epidemiological model are reported, but I couldn't see any information on K1, K2, and M0. The authors should state their interest in investigating the epidemiological model at this point.

***R: According with reviewer comment we added “of epidemiological parameters” at line 239.***

In line 299, the authors chose to refer the amplitude of parameters perturbations as CI, the confidence interval. I strongly recommend that the authors choose a different term for this range, as CI has already been widely misused in the literature.

***R: Following the reviewer’s comment we edited the ms by referring to the refer the amplitude of parameters perturbations as 97.5% quantiles (lines 299, 322, 323-324 and 326).***

In line 353, the grey boxplot visualising the posterior distribution seems to be missing in Figure 4.

***R: We thank the reviewer for highlighting the typo, we edited figure 4 according with reviewer’s comment.***

In the first paragraph of Discussion, the authors claim that they proved the importance of having a reliable and precise estimate of the bird recovery rate, but the presentation of this argument could be much improved. I believe this is a strong statement, and should be paraphrased.

***R: According to the reviewer’s suggestion, we rephrased the sentence by stating that we highlighted (instead of proved) the importance to have a good estimate of the recovery rate of birds (line 392).***

In line 487, it is not clear how the temperatures and mosquito densities in Lombardy always sustain WNV spread. Does this apply to every year, every time of the year, and even accounting for diurnal variability? I don't think such a strong statement is supported by the evidence presented.

***R: Following this comment we edited lines 485-488 to better explain what we meant with “temperatures and mosquito densities in Lombardy always sustain WNV spread”.***

In S1 Fig, H, the parameters analysed (f and pVB) do not match with the earlier description of model parameters. What is f in relation to f1 and f2? Is pVB the same as pMB?

***R We thank the reviewer for pointing this out, we edited S1 Fig H. We also edited line 187 in the main text to relate f to f1 and f2.***

The parameter "b" is not included in model description. Also the description of "p" is left out of Appendix S1. I believe the complete description of the model and equations, as claimed by the authors in lines 195-196, should include all such details and variations in parameters.

***R: We edited table A in S1 according with reviewer’s criticism.***

My final comment is that the manuscript seems to be written by multiple authors with different styles/experiences, and this seems to have disrupted coherence in certain parts. Other than this, I enjoyed reading this interesting manuscript.

***R: We thank the reviewer for the final positive comment about our work and we kindly appreciate their careful reading. As we propose a multidisciplinary work, equally accounting for needs and expertise of the different coauthors, ms coherence might have not been adequately considered and we apologize for that. We did our best to improve it in this version.***

**Reviewer #2**

I would like to thank the authors for their consideration of my comments and their work in editing the manuscript. I am now happy to recommend the work for publication. My only remaining comment is that I have selected **"No" in the data availability drop-down menu** because I don't think the model code is publically available, which in the name of open science I think it should be.

***R:*** ***We thank the reviewer for acknowledging the improvement of the ms obtained after the review process. We agree about the importance of open science and free exchange of scientific contents. Despite that the code itself was not written to a public audience, making it hard to use for an external user. To compensate for the lack of a publicly available code, we provided all data tables and equations describing the framework. Furthermore, codes can be obtained by contacting the corresponding author.***

**Reviewer #3**

(No Response)

--------------------

PLOS authors have the option to publish the peer review history of their article ([what does this mean?](https://journals.plos.org/plosntds/s/editorial-and-peer-review-process#_blank)). If published, this will include your full peer review and any attached files.


If you choose “no”, your identity will remain anonymous but your review may still be made public.


**Do you want your identity to be public for this peer review?** For information about this choice, including consent withdrawal, please see our [Privacy Policy](https://www.plos.org/privacy-policy).

Reviewer #1: No

Reviewer #2: Yes: David Ewing

Reviewer #3: No


Figure Files:
While revising your submission, please upload your figure files to the Preflight Analysis and Conversion Engine (PACE) digital diagnostic tool, https://pacev2.apexcovantage.com. PACE helps ensure that figures meet PLOS requirements. To use PACE, you must first register as a user. Then, login and navigate to the UPLOAD tab, where you will find detailed instructions on how to use the tool. If you encounter any issues or have any questions when using PACE, please email us at figures@plos.org.
 
Data Requirements:
Please note that, as a condition of publication, PLOS' data policy requires that you make available all data used to draw the conclusions outlined in your manuscript. Data must be deposited in an appropriate repository, included within the body of the manuscript, or uploaded as supporting information. This includes all numerical values that were used to generate graphs, histograms etc.. For an example see here: http://www.plosbiology.org/article/info%3Adoi%2F10.1371%2Fjournal.pbio.1001908#s5.
 
Reproducibility:
To enhance the reproducibility of your results, we recommend that you deposit your laboratory protocols in protocols.io, where a protocol can be assigned its own identifier (DOI) such that it can be cited independently in the future. Additionally, PLOS ONE offers an option to publish peer-reviewed clinical study protocols. Read more information on sharing protocols at https://plos.org/protocols?utm_medium=editorial-email&utm_source=authorletters&utm_campaign=protocols

__________________________________________________
In compliance with data protection regulations, you may request that we remove your personal registration details at any time. (Use the following URL: https://www.editorialmanager.com/pntd/login.asp?a=r). Please contact the publication office if you have any questions.
